# Weight-Reducing Effect of *Lactobacillus Plantarum* ZJUFT17 Isolated from Sourdough Ecosystem

**DOI:** 10.3390/nu12040977

**Published:** 2020-04-01

**Authors:** Tongjie Liu, Yang Li, Minjie Zhao, Qiufen Mo, Fengqin Feng

**Affiliations:** 1 College of Food Science and Engineering, Ocean University of China, Qingdao 266003, China; ltjpeak@126.com; 2 College of Biosystems Engineering and Food Science, National Engineering Laboratory of Intelligent Food Technology and Equipment, Zhejiang Key Laboratory for Agro-Food Processing, Zhejiang University, Hangzhou 310058, China; liyangstrel@163.com (Y.L.); minjiezhao@zju.edu.cn (M.Z.); mqfydws@163.com (Q.M.); 3 School of Food Science and Engineering, Qingdao Agricultural University, Qingdao 266109, China; 4 Ningbo Research Institute, Zhejiang University, Ningbo 315100, China

**Keywords:** probiotics, obesity, gut microbiota modulation, metabolic syndrome, sourdough

## Abstract

*Lactobacillus plantarum* ZJUFT17 (T17) is a potential probiotic bacterium isolated from Chinese traditional sourdough. The purpose of this study was to investigate its weight-reducing effects in mice fed a high-fat diet (HFD) and further to elucidate possible mechanisms. Male C57BL/6J mice fed HFD were given T17 (2–4 × 10^8^ cfu) intragastrically for 10 weeks. The results showed that the administration of T17 significantly suppressed HFD-induced body weight gain, alleviated HFD-induced increase in serum lipids and decreased energy intake. The serum levels of obesity-related metabolic signaling molecules, including insulin, adiponectin, lipopolysaccharide (LPS) and the cytokines interleukin (IL)-1β and tumor necrosis factor (TNF)-α, were markedly improved. The 16S rRNA gene sequencing revealed that T17 administration dramatically modulated the gut microbiota, suppressing pathogenic and pro-inflammatory microbes and stimulating the microbes favoring anti-obesity. The weight-reducing efficacy of T17 may be explained by its ability to ameliorate systemic inflammation and insulin resistance mediated by gut microbiota. This study revealed that T17 could ameliorate obesity and the concomitant metabolic syndrome in mice and that the lactic acid bacteria in the sourdough ecosystem may also possess anti-obesity/weight-reducing properties.

## 1. Introduction

Obesity is a pathological state characterized by abnormal or excessive fat accumulation, owing to a chronic imbalance between energy intake and energy expenditure [1]. It is a known risk factor for the development of a series of diseases, such as metabolic syndrome, type II diabetes, hypertension, hypercholesterolemia, cardiovascular disease, and some forms of cancer [2,3]. Starting in the early 1980s, the prevalence of obesity and overweight began to increase rapidly in high-income countries, and in 2015, two billion people were estimated to be affected by obesity worldwide [4]. Particularly, almost 340 million children and adolescents aged 5–19 years or, in another word, one in every five (18.4%) were overweight or obese globally in 2016 [5]. Obesity has now become a global health issue and the etiology of obesity involves both biological and environmental factors [2]. However, what needs to be emphasized is that an energy imbalance between calorie intake and expenditure is the fundamental cause of overweight and obesity, and globally, an increased consumption of energy-dense foods that contain high fat and sugar contents and a decrease in physical activity facilitates the prevalence of obesity [4].

Various methods have been implemented in overweight or obesity management and, in the last two decades, more and more attention has been paid to the role played by gut microbiota in the development and treatment of obesity [6,7]. It has been found that obesity is generally associated with an alteration in the composition of gut microbiota and, overall, people with obesity have less microbial diversity compared with lean people [6,8]. Furthermore, a large body of evidence has revealed the correlations between certain microbial communities and the occurrence of obesity, although the exact mechanisms remain obscure [9,10]. For instance, in some human studies and animal models, the Firmicutes/Bacteroidetes ratio was positively correlated with the obese phenotype [11] and, in deeper investigations, specific species of gut microbes have been targeted for their weight reduction potential. The *Akkermansia muciniphila*, a human intestinal mucin-degrading bacterium, was found to be strongly and negatively correlated with HFD-induced obesity [12]. Accumulative findings of the correlations between gut microbiota and obesity have raised the concept of microbial manipulation to combat or prevent obesity, particularly probiotics, defined as live microorganisms, which, when administered in adequate amounts, confer a health benefit on the host [13], are considered a useful tool to modulate gut microbiota therapeutically. Numerous probiotic strains, mainly involving lactobacilli and bifidobacteria, have been found to possess the ability to ameliorate obesity via regulating the gut microbiota composition [14]. Since the gut microbiota has close interactions with the host in terms of metabolic, signaling and immune activities, different probiotic strains exert weight reduction effects through different pathways [14]. Some probiotics could stimulate the secretion of certain hormones by enteroendocrine cells such as glucagon-like peptide-1 (GLP-1) and peptide YY (PYY) which could regulate appetite and food intake via interaction with specific receptors of neurons in the brain [15]. For example, the probiotic VSL#3 can modulate the gut microbiota–short–chain fatty acid (SCFA)–hormone axis, and promoted the secretion of the appetite-suppressing hormone GLP-1 via butyrate from altered gut microbiota [16]. Others may exert weight reduction effects through the inhibition of lipopolysaccharide (LPS) production and suppression of inflammatory pathways via the modulation of gut microbiota [17]. A myriad of research has indicated the potential of probiotics to tackle obesity, however, it is still not clear which microbes contribute more to the obesity etiology and how the probiotic strains interact with the gut microbiota. The gaps in our current knowledge open the platform for further research.

Sourdough has long been used as a starter in breadmaking throughout the world, and extensive research has indicated that lactic acid bacteria (LAB) is the predominant bacteria in the sourdough ecosystem [18]. There is no doubt that the sourdough LAB possesses excellent technological properties [19], however, their probiotic properties, especially in vivo condition, have been scarcely studied. In our previous research, the strain *Lactobacillus plantarum* ZJUFT17, isolated from Chinese traditional sourdough, was found to be helpful in suppressing body weight gain in mice supplemented with glycerol monolaurate [20]. In the present study, the efficacy of *Lactobacillus plantarum* ZJUFT17 in alleviating HFD-induced obesity and its possible mechanisms of action were investigated.

## 2. Materials and Methods

### 2.1. Preparation of L. Plantarum ZJUFT17

*L. plantarum* ZJUFT17 (T17) was isolated from sourdough in our previous research [21] and was deposited at China Center for Type Culture Collection with an accession number of CCTCC 2017342. The T17 strain was prepared following the previously described procedure [20]. Briefly, the T17 was cultured in de Man, Rogosa, and Sharpe (MRS) broth at 37 °C for 22 h, harvested by centrifugation (10,000×g for 5 min at 4 °C) and lyophilized with 5% (*w*/*v*) skim milk and 5% (*w*/*v*) lactose as protective agents. The microbial loads of the resulting lyophilized T17 was 2.03 × 10^11^ colony-forming units (cfu)/gram.

### 2.2. Animals and Treatment

Forty-five male C57BL/6J mice of 4 weeks old were purchased (Shanghai SLAC Laboratory Animal Co., Ltd, Shanghai, China) and housed at a specific pathogen-free (SPF) and air-conditioned room (22 ± 2 °C, 50% ± 10% relative humidity and a 12 h light/dark cycle) in the Laboratory Animal Research Center of Zhejiang Chinese Medical University. The experiment was approved by the animal ethical committee of the university (Resolution number ZSLL-2017-138). After one-week acclimation, the mice were divided into three groups (*n* = 15, three per cage) with free access to a mouse pellet diet and water. The NCD group was orally administered with 0.2 mL of sterile saline and received a normal chow diet (kcal% provided with 70% carbohydrates, 20% proteins and 10% fat; Shanghai SLAC Laboratory Animal Co., Ltd, Shanghai, China). The HFD group was orally administered 0.2 mL of sterile saline and received a high-fat diet (kcal% provided with 45% fat, 35% carbohydrates and 20% proteins; Jiangsu Medicience Co., Ltd, Jiangsu, China). A detailed diet composition is provided in Table 1. The HFT group was orally administered with 0.2 mL of sterile saline containing 2-4 × 10^8^ cfu of T17 and received a high-fat diet. All the animals in the three groups were treated daily in the morning for 10 weeks. The body weight of each animal and the food intake of each cage were recorded weekly. The body weight was expressed as a percentage compared to the initial body weight. After the 10-week treatment, the mice were fasted for 12 h, and then blood samples were collected from the retrobulbar, intraorbital, and capillary plexus. The mice were then sacrificed by cervical dislocation, and the liver, kidney, spleen and epididymal fat pad were excised, weighed and collected for subsequent analysis. 

### 2.3. Histological Analysis

The liver and epididymal fat pad samples were fixed in 10% (*v*/*v*) formalin/PBS (phosphate buffer solution) at room temperature and then embedded in paraffin for hematoxylin and eosin (H&E) staining. Then, the histochemical staining images were captured at × 100 or × 400 magnification using a microscope (Leica ICC50W, Wetzlar, Germany). For epididymal fat pad samples, the size and area of the stained adipocytes were analyzed using the Image Pro Plus software (Media Cybernetics Inc., Rockville, MD, USA). The size of the adipocytes was determined according to the previously described method [20].

### 2.4. Serum Biochemical Analysis

The levels of total triglycerides (TG), total cholesterol (T-CHO), high-density lipoprotein–cholesterol (HDL-C), low-density lipoprotein–cholesterol (LDL-C) and fasting glucose in serum were measured using corresponding kits (Nanjing Jiangcheng Bioengineering Institute, Nanjing, China). The serum level of insulin was determined using an ELISA kit (Elisalab, Wuhan, China). The atherogenic index and the homeostasis model assessment-insulin resistance index (HOMA-IR) was calculated (Atherogenic index = (T-CHO − HDL-C)/HDL-C, HOMA-IR = Fasting glucose (mM) × Fasting insulin (μU/mL )/22.5) according to previous publications [22,23]. The adiponectin and leptin levels were measured using ELISA kits from Multisciences (Lianke Biotech, Co., Ltd, Hangzhou, China). The serum LPS concentration was determined using a ToxinSensor Chromogenic Limulus Amebocyte Lysate (LAL) Endotoxin Assay Kit (GenScript, Piscataway, NJ, USA). Serum concentrations of interleukin (IL)-1β, IL-6 and IL-10, tumor necrosis factor (TNF)-α and transforming growth factor (TGF)-β were determined using ELISA kits from eBioscience. All the determinations were performed according to the manufacturer’s protocols and the absorbance was measured at different wavelengths, provided in the protocols using a microplate reader (Infinite M200Pro, Tecan, Austria). All the standards used were run at least in duplicates, and their intra-assay coefficients of variability ranged from 1.93% to 13.63%.

### 2.5. Gut Microbiota Analysis

Genomic DNA was directly extracted from fecal samples of the mice treated for 10 weeks (*n* = 9-10 per group) using a QIAamp DNA Stool Mini Kit (QIAGEN, Venlo, Netherlands) following the manufacturer’s protocol. Paired-end sequencing of the 16S rRNA gene targeting the V3-V4 hypervariable region was performed using the Illumina HiSeq2500 platform (Realbio Technology Inc., Shanghai, China). The post-sequencing data analysis was performed according to our previous work [22]. LDA effect size (LEfSe) was carried out based on linear discriminant analysis to estimate the influence of the abundance of each species on the difference between groups, and find out the microbial communities or species that have a significant impact on sample partition, using an LEfSe platform [24]. The correlation analysis between gut microbiota and metabolic parameters in mice was performed using the R software.

### 2.6. Statistical Analysis

Statistical analysis was performed via one-way analysis of variance (ANOVA) followed by Tukey’s test using the software GraphPad Prism 6 (GraphPad Software, La Jolla, CA). The data were expressed as mean ± SEM (standard error of mean). *P* < 0.05 was considered statistically significant.

## 3. Results

### 3.1. L. Plantarum ZJUFT17 Alleviated HFD-Induced Obesity and Lipid Accumulation

**Figure 1:**
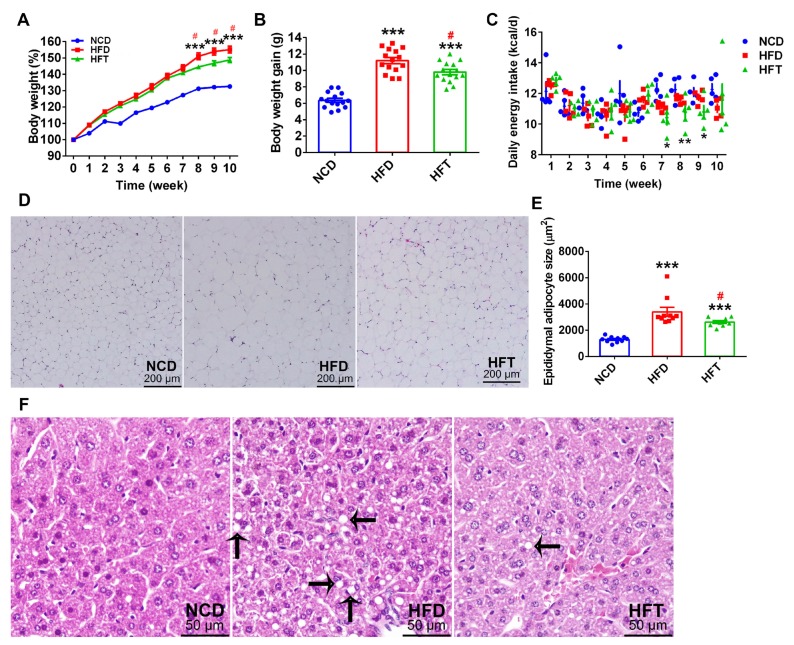
T17 administration suppressed body weight gain and lipid accumulation induced by high-fat diet (HFD). (**A**) body weight changes in 10 weeks, (**B**) body weight gain in the 10 weeks, (**C**) average daily energy intake per cage of the three groups in 10 weeks, (**D**) the H&E staining image of epididymal adipose tissue sections, (**E**) the sizes of stained epididymal adipocytes in the three groups, (**F**) hepatic lipid accumulation revealed by H&E staining and indicated with arrows. Data are expressed as mean ± SEM. Values marked with * are significantly different compared with the normal chow diet (NCD) group and values marked with # are significantly different compared with HFD group (* *p* < 0.05, ** *p* < 0.01, *** *p* < 0.001, # *p* < 0.05).

As shown in Figure 1, the percent body weight of the mice a fed high-fat diet (HFD and HFT group) increased more quickly than that of mice fed a normal chow diet (NCD group) and, consequently, the mice in HFD and HFT groups showed a significantly higher body weight gain than those in the NCD group. It was worth noting that body weight difference between the HFD and HFT groups occurred after 7 weeks of treatment and finally, the HFT group showed a significantly lower body weight gain compared with the HFD group after the 10-week treatment. As shown in Figure 1C, the HFT group exhibited a significantly decreased energy intake during week 7–9. Although significant differences were not observed regarding the weight of the liver and epididymal fat pad between HFD and HFT groups (Table 2), the H&E staining showed that the HFT group had a significantly smaller size of adipocyte in epididymal adipose tissue and less multi-locular lipid droplets in the liver (Figure 1D–F). 

### 3.2. L. Plantarum ZJUFT17 Improved Serum Lipid Profiles

**Figure 2:**
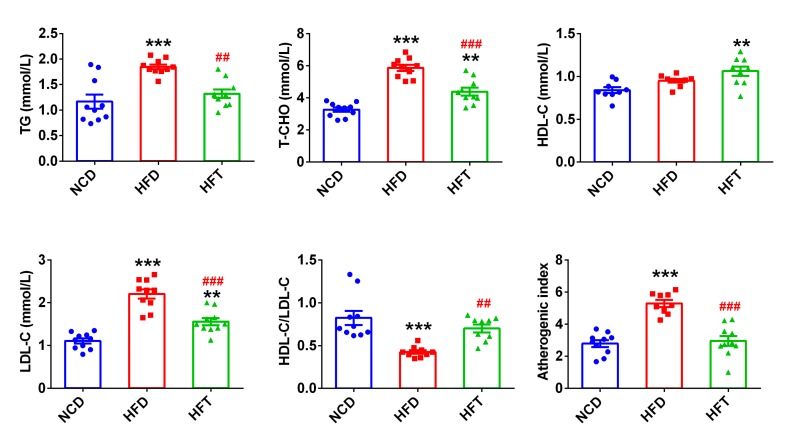
The effects of T17 on serum lipids profiles in mice fed HFD for 10 weeks. Data are expressed as mean ± SEM. Values marked with * are significantly different compared with NCD group and values marked with # are significantly different compared with HFD group (** or ## *p* < 0.01, *** or ### *p* < 0.001).

HFD markedly elevated the serum levels of TG, T-CHO and LDL-C, whereas the administration of T17 significantly attenuated the elevation caused by HFD (Figure 2). Noticeably, the HFT group showed a significantly higher level of HDL-C than the NCD group, and consequently, the HDL-C/LDL-C value in the HFD group was significantly lower than that in the NCD and HFT groups, and the inverse was true for the atherogenic index.

### 3.3. L. Plantarum ZJUFT17 Modulated the Serum Levels of Insulin and Adipokines

**Figure 3:**
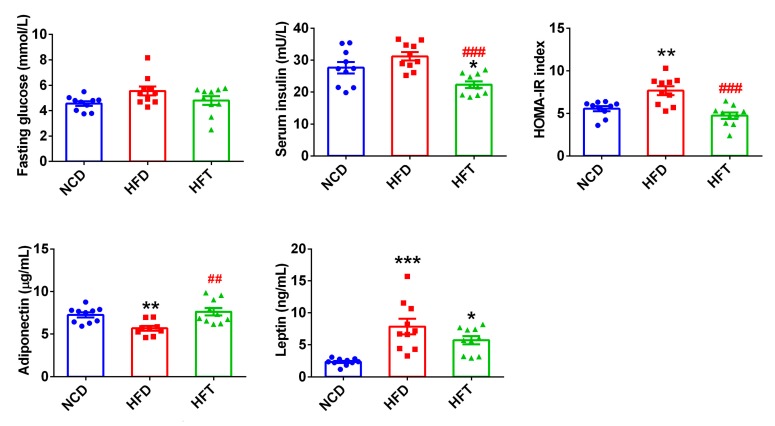
Administration of T17 attenuated insulin resistance and modulated adipokines. Data are expressed as mean ± SEM. Values marked with * are significantly different compared with NCD group and values marked with # are significantly different compared wih HFD group (* *p* < 0.05, ** or ## *p* < 0.01, *** or ### *p* < 0.001).

There was no significant difference concerning the fasting plasma glucose levels in three groups (Figure 3). However, the fasting plasma glucose level in the HFD group was higher, though not significantly, than that in the NCD group (*p* = 0.073). The mice administered with T17 had a significantly lower level of serum insulin and a significantly reduced HOMA-IR index than those in NCD and HFD groups (Figure 3). As for the adipokines, the consumption of HFD significantly impacted the serum levels of adiponectin and leptin. Treatment with T17 significantly restored the loss of the adiponectin caused by HFD, and tended to decrease, though not significantly, the high leptin level induced by HFD (5.73 vs. 7.86).

### 3.4. L. Plantarum ZJUFT17 Attenuated Systemic Inflammation Induced by HFD

**Figure 4:**
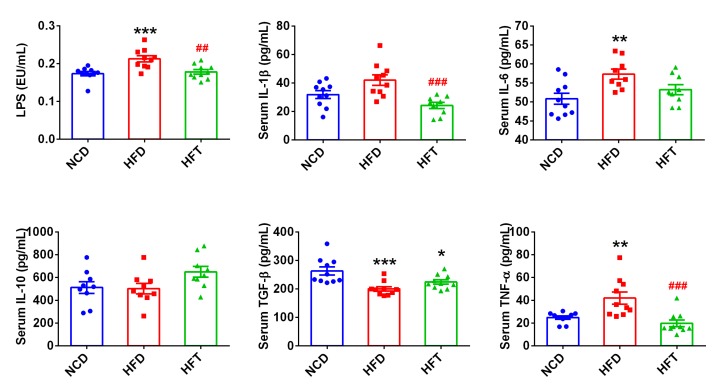
T17 administration significantly attenuated increased the circulating level of LPS and pro-inflammatory cytokines induced by HFD. Data are expressed as mean ± SEM. Values marked with * are significantly different compared with NCD group and values marked with # are significantly different compared with HFD group (* *p* < 0.05, ** or ## *p* < 0.01, *** or ### *p* < 0.001).

As shown in Figure 4, the HFD group had significantly higher levels of LPS and pro-inflammatory cytokines, such as IL-6, TNF-α and IL-1β (*p* = 0.0549), than the NCD group. T17 administration (HFT group) significantly decreased the levels of LPS, IL-1β, and TNF-α. In addition, the two anti-inflammatory cytokines, TGF-β and IL-10, were both higher, although not significantly, in the HFT group than in the HFD group.

### 3.5. High-Fat Diet and L. Plantarum ZJUFT17 Administration Altered Gut Microbiota

**Figure 5:**
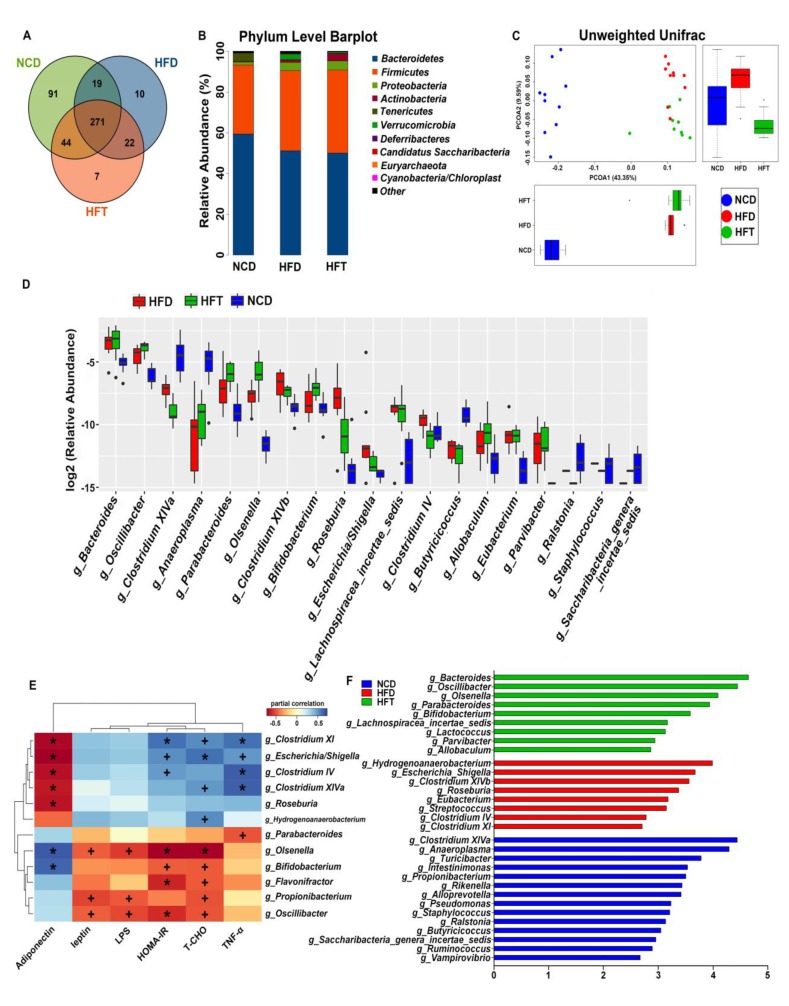
HFD and T17 administration altered the diversity and composition of gut microbiota. (**A**) Venn Diagram of operational taxonomic units (OTUs), (**B**) bar plot of microbial communities at the phylum level, (**C**) PCoA plot based on unweighted UniFrac distances, (**D**) boxplot based on the significantly altered genera, (**E**) spearman’s correlations between key microbes and metabolic parameters, (**F**) the discovered genera (biomarkers) using linear discriminant analysis effect size with 2.5 as the LDA score threshold. (+ *p* < 0.05, * *p* < 0.01).

The results of the high throughput sequencing of the fecal samples were listed in Figure 5. As shown in the venn diagram, the NCD group possessed the most operational taxonomic units (OTUs) followed by the HFT group, in accordance with the α-diversity indices of the three groups, where the NCD group showed the most diversity (Table 3). The principal coordinate analysis (PCoA), based on unweighted UniFrac distances, demonstrated that the NCD group was markedly separated from the HFD and HFT groups along the PCoA1 axis; meanwhile, the HFD and HFT group could be separated along the PCoA2 axis. The classification abundance analysis showed that the average Firmicutes/Bacteroidetes ratio was non-significantly increased in the HFD-treated groups, being 0.64, 0.86 and 0.93 in NCD, HFD and HFT, respectively. However, considerable differences in relative abundance could be seen among the groups at the genus level, with 19 genera significantly altered. The consumption of an HFD significantly increased the relative abundance of *Bacteroides*, *Oscillibacter*, *Parabacteroides*, *Olsenella*, *Clostridium* XIVb, *Roseburia*, *Escherichia*/*Shigella*, *Lachnospiracea incertae sedis*, *Clostridium* IV, *Eubacterium* and *Parvibacter*, and significantly decreased the relative abundance of *Clostridium* XIVa, *Anaeroplasma*, *Butyricicoccus*, *Ralstonia* and Candidatus *Saccharibacteria genera incertae sedis*. Meanwhile, a remarkable distinction between the HFT group and the other two could be seen at the genus level. The administration of T17 dramatically elevated the relative abundance of *Parabacteroides*, *Olsenella* and *Bifidobacterium* and reduced the relative abundance of *Ralstonia* compared with the HFD and NCD groups. Noticeably, significant differences could also be observed between the HFT and HFD groups regarding the relative abundance of genera *Oscillibacter*, *Olsenella, Clostridium* XIVb, *Clostridium* IV and *Clostridium* XI. Particularly, *Bifidobacterium* and *Lactococcus* were biomarkers in the HFT group, as revealed by LEfSe analysis, while *Escherichia*/*Shigella* was relatively high in the HFD group. To further compare the HFD and HFT group, a heatmap was generated to demonstrate the correlations between their biomarker microbes and the metabolic indicators related to obesity/metabolic syndrome. Generally, the indicator genera in the HFT group such as *Bifidobacterium* and *Olsenella* correlated negatively, to varying degrees, with LPS, leptin, T-CHO, TNF-α level and HOMA-IR value, and were positively correlated with adiponectin level. On the contrary, the biomarkers in HFD such as *Escherichia*/*Shigella* and *Clostridium* XI correlated positively with HOMA-IR value and the levels of T-CHO and TNF-α, and were correlated negatively with adiponectin level (Figure 5E).

## 4. Discussion

A high-fat diet (HFD) is a significant contributor to the prevalence of obesity and obesity-related metabolic disorders. Accumulating evidence has shown that probiotics’ administration could help to suppress HFD-induced obesity and metabolic symptoms, which is a clustering of at least three of the five following medical conditions: central obesity, high blood pressure, high blood sugar, high serum TG, and low serum HDL [25]. In this study, the mice in the HFT group exhibited an average of a 12.5% decrease in body weight gain compared with their HFD counterparts during the 10-week treatment, and the HFT did decrease food intake from week 7–9 which may thus contribute to a decrease in body weight gain. The different body weight gain between the HFD and HFT groups indicated that T17 was able to attenuate HFD-induced body weight gain, showing a weight-reducing efficacy. Generally, the increase in fat mass or body weight in obese hosts was characterized by increased intracellular lipids content, a greater adipocyte size and increased numbers of adipocytes [1]. In this research, T17 decreased lipid accumulation in epididymal adipose tissue and in the liver induced by an HFD, which may explain, at least partly, its weight-reducing efficacy. Furthermore, it was worth noting that the administration of T17 significantly improved serum lipid profiles and contributed to a decreased atherogenic index. Taking the above into consideration, T17 showed the potential to ameliorate metabolic syndrome caused by HFD. In addition, metabolic syndrome is considered to increase the risk of cardiovascular disease and type 2 diabetes [26], and, therefore, T17 administration may also be helpful in the prevention of these two diseases.

Obesity/metabolic syndrome is always associated with insulin resistance, which is defined as the impaired sensitivity of organs/tissues such as the liver, skeletal muscle and adipose tissues to the action of insulin and can be induced by HFD [27]. Insulin resistance could decrease lipolysis rates in adipose tissue, which could further result in an increased level of TG in plasma [28]. In this study, the HOMA-IR index in the HFD group was significantly increased and, correspondingly, a significant elevation of TG was observed as well as an elevation of the T-CHO and LDL-C levels. Meanwhile, adiponectin, an adipose tissue-secreted endogenous insulin sensitizer, was significantly reduced in the HFD-fed mice, in line with the occurrence of insulin resistance, which has been reported in obese mice or humans [29,30]. The administration of T17, however, significantly alleviated insulin resistance and decreased the serum levels of TG, T-CHO and LDL-C in HFD-fed mice, which may be partly explained by the significant increase in adiponectin, assumedly due to the reduction in adipocyte size in accordance with previous studies [31]. Adiponectin is known to play an important role in regulating energy homeostasis, and the improvement in adiponectin as a result of T17 feeding may be linked to the regulation of lipid metabolism, leading to a balanced serum lipid profile [32]. Another hormone synthesized and secreted by adipose tissue, leptin, is produced in proportion to fat stores and exerts negative feedback effects on energy intake to permit energy expenditure. However, paradoxically, elevated levels of leptin in obese hosts, especially diet-induced obesity, may indicate the development of leptin resistance, which may play a role in the development of obesity [33]. The mice in the HFD group displayed significantly elevated circulating leptin levels compared to the mice in the NCD group, implying that leptin resistance occurred in the HFD-induced obese mice. Compared with the HFD group, the HFT group showed a reduced average of leptin (non-significantly, 5.73 vs. 7.86), which was in line with their lower lipid accumulation in adipose tissue and smaller adipocyte size, and may suggest an amelioration of leptin resistance.

Obesity is usually accompanied by a systemic low-grade inflammation featuring elevated circulating levels of pro-inflammatory cytokines [34]. The HFD-induced increase in plasma lipopolysaccharide (LPS) was found to serve as a triggering factor in the occurrence of inflammation, insulin resistance and obesity [35]. In the present study, the mice fed an HFD showed a significantly increased circulating level of LPS and, correspondingly, their serum levels of two pro-inflammatory cytokines, IL-6 and TNF-α, were significantly elevated, demonstrating the occurrence of a low-grade inflammatory status [14]. The results showed that T17 administration could significantly decrease the circulating LPS level and simultaneously reduce the serum levels of IL-1β and TNF-α, indicating that T17 could attenuate the inflammation in obese mice caused by HFD. It has been reported that elevated LPS and the resulting pro-inflammatory cytokines released were identified as triggering factors for insulin resistance [35]. Therefore, the amelioration of the systemic inflammation by T17 may also be responsible for its efficacy in alleviating insulin resistance, in addition to the stimulation of adiponectin secretion [34].

Obesity generally occurs with an alteration of the composition of gut microbiota characterized by reduced diversity and species richness [6], which was confirmed in this study. The obese mice fed an HFD showed a marked change in gut microbiota in comparison with the mice fed a normal chow diet. Extensive research has revealed that probiotics could ameliorate obesity/metabolic syndrome via the modulation of gut microbiota, though the mechanism of action of probiotics is obscure [14,25,36]. The proposed mechanisms of action include antagonistic effects on pathogen growth and competitive adhesion to the intestinal mucosa (antimicrobial activity), increasing intestinal mucus layer and reducing intestinal permeability (barrier function), and modulation of the intestinal immunity (immunomodulation) [9]. The results of our study showed that the administration of T17 led to a different composition in the gut microbiota compared with the mice in HFD and NCD groups, which confirmed the effect of T17 on gut microbiota modulation, as reported in our previous research [20]. In this study, T17 may exert its weight-reducing efficacy via gut microbiota modulation, or, more specifically, by altering the relative abundance of inflammation-related microbes. The genera *Clostridium* XI, *Clostridium* IV and *Escherichia*/*Shigella*, the biomarkers of the HFD group based on the LEfSe analysis, were found to be positively correlated with the proinflammatory cytokine TNF-α, indicating that these genera may have a proinflammatory role during obesity development. Particularly, the increase in the genera *Clostridium* XI, which contains the pathogenic species *Clostridium difficile*, may be considered as a risk for health [37]. The reduction in these genera in the HFT group suggested that T17 might alleviate the inflammation by suppressing intestinal pathogens. Meanwhile, T17 may also favor the growth of anti-inflammatory microbes, such as the genera *Parabacteroides* [38], which was a biomarker of the HFT group and negatively correlated with TNF-α. Recent studies have shown that *Parabacteroides* spp. has an anti-obesity effect and is associated with reduced inflammation [39,40]. Furthermore, the administration of T17 significantly increased in the relative abundance of *Bifidobacterium*, which plays a prominent role in inhibiting LPS-induced inflammation [34]. It has been reported that the feeding of *Bifidobacterium* spp. could reverse metabolic endotoxemia, a status where the permeability of the intestine increases and allows the translocation of microbiome-derived LPS to the bloodstream, resulting in a two- to threefold increase in its serum concentrations, and improve gut integrity in mice [41,42]. In this study, however, the negative correlation between the *Bifidobacterium* and LPS was not significant, thus whether the increase in *Bifidobacterium* contributed to the decrease in serum LPS in the HFT group is unclear. Nevertheless, another two biomarkers of HFT group, *Olsenella* and *Oscillibacter*, were significantly negatively correlated with LPS, and the underlying mechanism merits further investigation. Meanwhile, it is worth noting that the genera *Bifidobacterium*, *Olsenella* and *Oscillibacter* were significantly negatively correlated with HOMA-IR, while the former two were also significantly positively correlated with adiponectin. It has been found that the oral administration of *Bifidobacterium* spp. could improve insulin resistance and induce adiponectin [43]. Therefore, the alteration of microbes by T17 administration may also contribute to the improvement in insulin resistance and adiponectin secretion. Apart from the above microbes, some genera, the abundance of which remarkably increased in the T17 group, may also account for the weight-reducing effect of T17. For instance, some species of *Bacteroides* have been found to possess an anti-obesity ability [44]. Therefore, T17 may exert its weight-reducing effect in this study by stimulating the relevant microbes. 

## 5. Conclusions

This study revealed that the administration of *Lactobacillus plantarum* ZJUFT17 could ameliorate HFD-induced body weight gain and improve the serum lipid profiles in mice, supposedly by alleviating systemic inflammation and insulin resistance, which may be mediated by gut microbiota modulation. *Lactobacillus plantarum* ZJUFT17 could markedly suppress some pro-inflammatory microbes such as *Clostridium* XI and *Escherichia*/*Shigella*, and stimulate other microbes that correlated positively with an anti-obesity or anti-inflammatory effects, such as *Bacteroides, Olsenella*, *Oscillibacter, Bifidobacterium* and *Parabacteroides*. However, the underlying mechanism of action of *Lactobacillus plantarum* ZJUFT17 on weight reduction and gut microbiota modulation still needs further exploration. Our study, however, showed that *Lactobacillus plantarum* ZJUFT17 may be used in the production of remedial and functional foods for obesity/metabolic syndrome management and that sourdough can serve as a reservoir of probiotics.

## Figures and Tables

**Table 1:** Compositions of experimental diets.

Ingredients (g/100 g Diet)	Normal Chow Diet	High Fat Diet
Casein	18.96	23.31
L-Cystine	0.28	0.35
Corn Starch	29.86	8.48
Maltodextrin	3.32	11.65
Sucrose	33.17	20.14
Cellulose	4.74	5.83
Soybean Oil	2.37	2.91
Lard	1.90	20.68
Mineral Mix	2.68	3.31
Potassium Citrate, 1 H_2_O	1.56	1.92
Vitamin Mix	0.95	1.16
Choline Bitartrate	0.19	0.23
**Calories supplementation (kcal %)**		
Proteins	20	20
Carbohydrates	70	35
Fats	10	45
**Total calories (kcal/100 g diet)**	385	473

**Table 2:** The effects of T17 on the weight of organs and adipose tissue in male C57BL/6 mice.

Organs and Tissues	Weight of Organs and Adipose Tissue (% of Final Body Weight)		
	NCD	HFD	HFT
Liver	3.57 ± 0.28 ^a^	3.26 ± 0.23 ^b^	3.22 ± 0.16 ^b^
Kidney	1.38 ± 0.06 ^a^	1.19 ± 0.07 ^b^	1.19 ± 0.08 ^b^
Spleen	0.26 ± 0.03	0.25 ± 0.03	0.23 ± 0.02
Epididymal fat	1.71 ± 0.31 ^a^	4.12 ± 0.78 ^b^	3.75 ± 0.85 ^b^

Data were expressed as mean ± SEM (*n* = 10). Different lowercase letters indicated significant differences between three groups.

**Table 3:** The α-diversity indices of gut microbiota in C57BL/6 mice.

α-Diversity	Mean Value			*p* Value
	NCD	HFD	HFT	
Chao1	300.2 ^a^	227.3 ^b^	236.0 ^b^	0.00019
Observed_species	263.7 ^a^	202.2 ^b^	213.3 ^b^	0.00015
PD_whole_tree	18.03 ^a^	14.49 ^b^	15.26 ^b^	0.00007
Shannon	5.568 ^a^	5.104 ^b^	5.158 ^b^	0.01402
Simpson	0.9576 ^a^	0.9404 ^b^	0.9444 ^ab^	0.01292
Goods_coverage	0.9983 ^a^	0.9989 ^b^	0.9987 ^b^	0.00207

Different letters within the same row indicate significant difference (*p* < 0.05).
